# Supercritical CO_2_ Extraction of Bioactive Compounds from Corn Grains (*Zea mays* L., Hybrid *Pri-15-7-16*) with Metabolomic Profiling and Confocal Laser Microscopy

**DOI:** 10.3390/plants14060913

**Published:** 2025-03-14

**Authors:** Mayya P. Razgonova, Pavel A. Shinkaruk, Anastasiia A. Maksimenko, Anna B. Podvolotskaya, Liudmila A. Tekutyeva

**Affiliations:** 1 N.I. Vavilov All-Russian Institute of Plant Genetic Resources, 42, 44 Bolshaya Morskaya, 190031 Saint Petersburg, Russia; 2 Institute of Biotechnology, Bioengineering and Food Systems, Advanced Engineering School, Far Eastern Federal University, 10 Ajax Bay, Russky Island, 690922 Vladivostok, Russia; shinkaruk.pa@dvfu.ru (P.A.S.); maksimenko.aal@dvfu.ru (A.A.M.); podvolotckaia.ab@dvfu.ru (A.B.P.); tekuteva.la@dvfu.ru (L.A.T.)

**Keywords:** corn grains, *Zea mays* L., CO_2_ extraction, polyphenolic compounds, tandem mass spectrometry, confocal laser microscopy, animal feed

## Abstract

This study aimed to optimize supercritical CO_2_ extraction conditions, analyze bioactive compounds, and visualize their distribution in corn grains (*Zea mays* L., hybrid *Pri-15-7-16*). The optimal extraction conditions were identified as a pressure of 200 bar and a temperature of 55 °C, yielding 2.2 mg/g of bioactive compounds. The distribution of autofluorescent compounds within corn grain tissues was visualized using confocal laser scanning microscopy. Image analysis showed that the pericarp and aleurone layer cell walls were rich in autofluorescent compounds, while the endosperm cell walls exhibited low autofluorescence. Metabolomic analysis, combining high-performance liquid chromatography and mass spectrometry, identified 44 compounds in the extracts, including 30 polyphenolic compounds from subgroups such as polyphenolic acids, flavones, flavan-3-ols, flavonols, and anthocyanidins as well as 14 compounds from other chemical groups, including amino acids and fatty acids.

## 1. Introduction

Vitamin D_3_ plays a crucial role in maintaining animal health by regulating calcium and phosphorus metabolism, bone formation, and immune function [1,2,3]. In dogs and cats, vitamin D_3_ is particularly important because they cannot synthesize it through sunlight exposure and must obtain it from their diet [2]. However, ensuring adequate vitamin D_3_ levels in animal feeds is challenging due to its sensitivity to environmental factors and low bioavailability [4,5].

Encapsulation and delivery systems for vitamin D_3_ are gaining importance in animal nutrition research [6]. These technologies protect the vitamin from environmental degradation, control its release, and enhance its bioavailability [7,8]. For instance, nanostructured lipid carriers have been developed for encapsulating vitamin D_3_, demonstrating increased stability and protection during digestion [6].

Different delivery systems significantly affect vitamin D_3_ bioavailability. A study comparing microencapsulated, micellar, and oil-based vitamin D_3_ supplements in laboratory rats showed that microencapsulated and oil-based forms were more bioavailable than micellar forms [4]. Specifically, the microencapsulated form exhibited a prolonged effect, maintaining elevated vitamin D_3_ levels for 14 days post-supplementation. Additionally, a study on dairy goats at the end of lactation showed that vitamin D_3_ encapsulated in sulfur-saturated coacervates of bovine lactoferrin–alginate complexes increased serum levels of 25-hydroxyvitamin D_3_ [25-(OH)-D_3_], lactoferrin, immunoglobulin A, and the immunomodulatory cytokine INF-γ [1]. Incorporating bioactive compounds of plant origin into delivery systems can further enhance vitamin D_3_ effectiveness through synergistic effects [9].

The use of bioactive plant compounds, particularly polyphenols, offers a promising strategy to improve vitamin stability and efficacy in animal diets. Polyphenols have garnered significant attention due to their antioxidant, anti-inflammatory, and antimicrobial properties [10,11,12,13,14]. They enhance nutrient stability and bioavailability by acting as natural preservatives, potentially protecting vitamin D_3_ from degradation during feed processing and storage, thereby improving its effectiveness in animal husbandry.

Moreover, plant polyphenols serve as natural antioxidants in animal feeds, promoting animal health and improving product quality. These compounds present viable alternatives to synthetic antioxidants in livestock feeding. Their inclusion in animal nutrition has been linked to improved growth performance, enhanced immune function, and better meat quality [15].

Among cereals, corn grains stand out for their exceptionally high polyphenol content, reaching 15.55 μmol/g. They also exhibit the highest overall antioxidant activity among common cereals such as rice, wheat, and oats, making them ideal for phytochemical and metabolomic studies [10,16,17].

Corn-derived phenolic compounds possess multifaceted protective properties, including radical scavenging, metal binding, and antioxidant activity. By reducing oxidative stress caused by free radical imbalances, polyphenols play a vital role in maintaining cellular health [10]. The antioxidant activity of corn polyphenols varies by variety, with colored corn containing significantly more polyphenols and anthocyanins than white or yellow corn. These pigmented varieties exhibit higher antioxidant activity due to their greater content of bioactive compounds [18,19,20,21]. A study by Feregrino-Pérez et al. (2024) [22] investigated the antioxidant activity and polyphenol content in corn grains and ears, revealing that corn ears generally contain higher levels of antioxidants and phenolic compounds than grains. Notably, the antioxidant capacity of purple corn ears is four to seven times higher than that of grain samples. Both grains and ears contain various phenolic compounds, including flavonoids, anthocyanins, and phenolic acids, with purple corn being particularly rich in anthocyanins [22].

The mechanisms by which phenolic compounds combat oxidative stress are diverse. They can directly neutralize reactive oxygen species, prevent their formation, or enhance the body’s innate antioxidant defense mechanisms to restore redox balance. The effectiveness of polyphenols in scavenging free radicals is attributed to their molecular structure, particularly the presence of aromatic rings and an extensive conjugated system rich in hydroxyl groups. Both the quantity and spatial arrangement of these hydroxyl groups significantly influence the antioxidant activity of these compounds [23,24].

Flavonoids function as exogenous antioxidants by neutralizing free radicals through various mechanisms, including enzyme inhibition and modulation of signaling pathways. Their antioxidant activity depends on their molecular structure, particularly the arrangement and quantity of hydroxyl groups. The antioxidant capacity of phenolic compounds follows the following order: simple phenolic acids < hydroxycinnamic acids < flavonols < flavan-3-ols < procyanidin dimers. Hydroxycinnamic acids exhibit stronger antioxidant effects than hydroxybenzoic acids due to their structural characteristics [10].

The application of supercritical fluid extraction for isolating phenolic compounds from natural plant materials represents a promising strategy in modern biotechnology. This method, particularly when utilizing CO_2_, serves as an effective and eco-friendly technique for extracting biologically active substances. Compared to traditional approaches such as Soxhlet extraction, supercritical CO_2_ offers several notable advantages: lower density and viscosity, higher diffusivity, and minimal environmental impact. A key benefit of this technique is its capacity for precise control of process parameters. By adjusting pressure and temperature, researchers can significantly enhance both the efficiency and selectivity of the extraction process, which is particularly important when dealing with complex plant matrices. Furthermore, a major advantage is the ability to obtain a solvent-free final product, as CO_2_ can be easily removed during depressurization, ensuring high extract purity [14,25,26,27,28,29,30,31,32,33,34].

These unique characteristics make supercritical CO_2_ extraction an especially attractive method for isolating bioactive compounds, including phenolic complexes, across various fields—from the food industry to pharmaceuticals. This approach opens new opportunities for developing innovative products with enhanced functional properties [25,26,28,29,30,35,36,37].

Screening various plant sources to identify valuable bioactive compounds, particularly polyphenolic complexes, for the production of vitamin biocomplexes intended for animal feed is one of the main research focuses. By studying natural antioxidants and developing innovative encapsulation methods, we aim to enhance the stability, bioavailability, and overall effectiveness of vitamin supplements in animal diets. This comprehensive approach has the potential to lead to the development of more effective vitamin formulations for livestock production, ultimately contributing to improved health and productivity in agricultural animals.

The objectives of this study were (1) to optimize supercritical CO_2_ extraction conditions for extracting bioactive compounds from corn grains (*Zea mays* L., hybrid *Pri-15-7-16*); (2) to analyze bioactive compounds in the extracts using high-performance liquid chromatography and mass spectrometry; and (3) to visualize the localization of phytochemical compounds in corn grain tissues using confocal laser scanning microscopy.

## 2. Results

### 2.1. Extraction of Bioactive Compounds

The extraction of bioactive compounds from corn grains was performed using two methods: maceration and supercritical CO_2_ extraction. Table 1 presents the yield of bioactive compounds extracted from corn grains (*Zea mays* L., hybrid *Pri-15-7-16*) under different temperature and pressure conditions during supercritical CO_2_ extraction. The temperature ranged from 31 °C to 60 °C, while the pressure varied from 50 to 250 bar. Yields ranged from a minimum of 0.30 mg/g (at 50 bar and 31 °C) to a maximum of 2.20 mg/g (at 200 bar and 55 °C).

**Figure 1:**
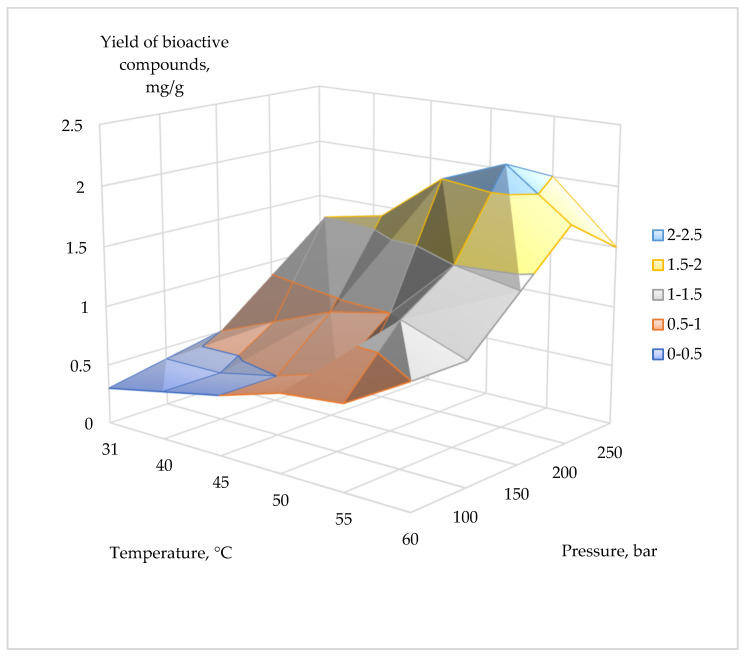
Three-dimensional graph of total yield of bioactive compounds during supercritical CO_2_ extraction of corn grains (*Zea mays* L., hybrid *Pri-15-7-16*) depending on pressure and temperature.

In general, increasing pressure and temperature resulted in higher yields, with a peak yield of 2.20 mg/g observed at 200 bar and 55 °C, which was identified as the optimal extraction condition. However, yields slightly decreased at the highest temperature and pressure levels. Additionally, yields of 2.00 mg/g were recorded at both 200 bar/50 °C and 250 bar/55 °C (Table 1, Figure 1).

### 2.2. Identification of Bioactive Compounds

The identification of chemical compounds in extracts from corn grains (*Zea mays* L.) was conducted using metabolomic analysis, combining high-performance liquid chromatography and mass spectrometry methods. In the supercritical CO_2_ extracts of corn grains, 44 compounds were identified—30 belonging to the polyphenol group and 14 to other chemical groups. All identified compounds, along with their chemical formulas, molar masses, ion adducts ([M−H]^−^ and [M+H]^+^), and MS/MS fragmentation data (1st, 2nd, and 3rd order), are presented in Table A1 (Appendix A).

### 2.3. Visual Localization of Bioactive Compounds 

**Figure 2:**
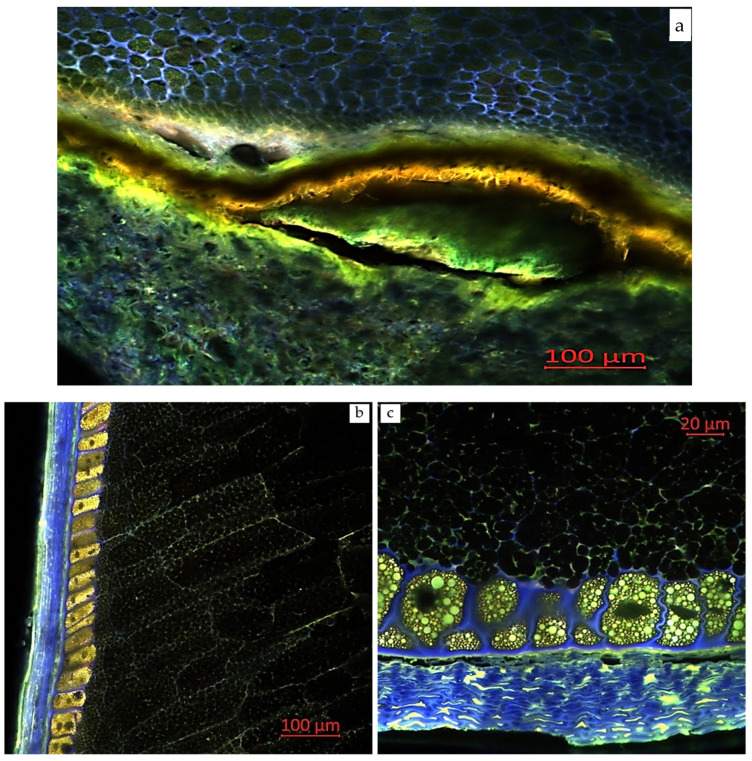
Multispectral image of corn grains (*Zea mays* L., hybrid *Pri-15-7-16*): (**a**) longitudinal section of the seed base (near the attachment point to the cob), 20× magnification; (**b**) longitudinal section, 20× magnification; and (**c**) transverse section of the distal edge of the grain, 63× magnification. Excitation with a UV laser (405 nm) with emission in the range of 400–475 nm (blue); and excitation with a blue laser (488 nm) with emission in the ranges of 410–545 nm (green) and 575–617 nm (yellow).

**Figure 3:**
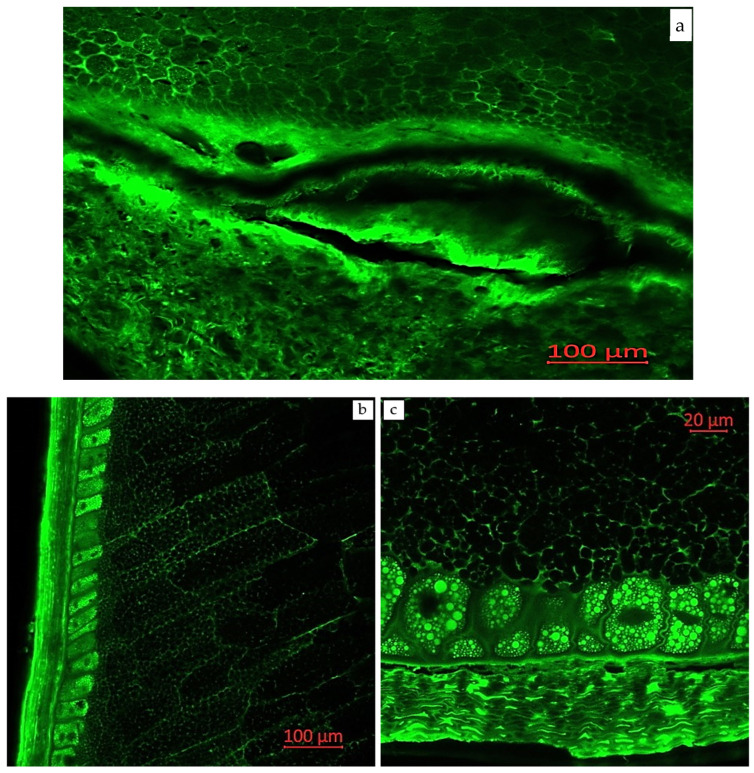
Monospectral images of corn grains (*Zea mays* L., hybrid *Pri-15-7-16*): (**a**) longitudinal section of the seed base (near the attachment point to the cob), 20× magnification; (**b**) longitudinal section, 20× magnification; and (**c**) transverse section of the distal edge of the grain, 63× magnification. Excitation with a blue laser (488 nm) with emission in the range of 410–545 nm (green autofluorescence).

**Figure 4:**
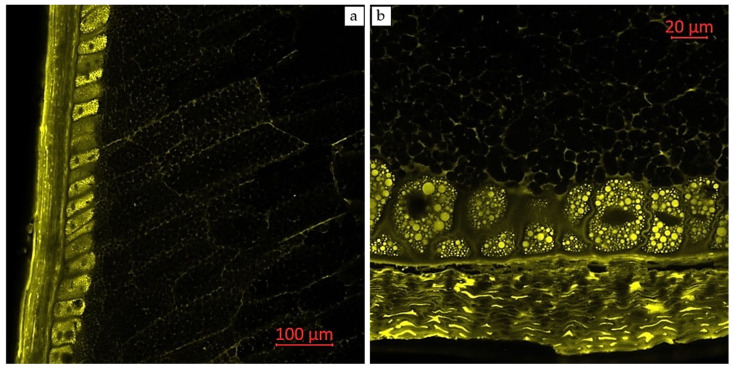
Monospectral images of corn grains (*Zea mays* L., hybrid *Pri-15-7-16*): (**a**) longitudinal section, 20× magnification; and (**b**) transverse section of the distal edge of the grain, 63× magnification. Excitation with a blue laser (488 nm) with emission in the range of 575–617 nm (yellow autofluorescence).

**Figure 5:**
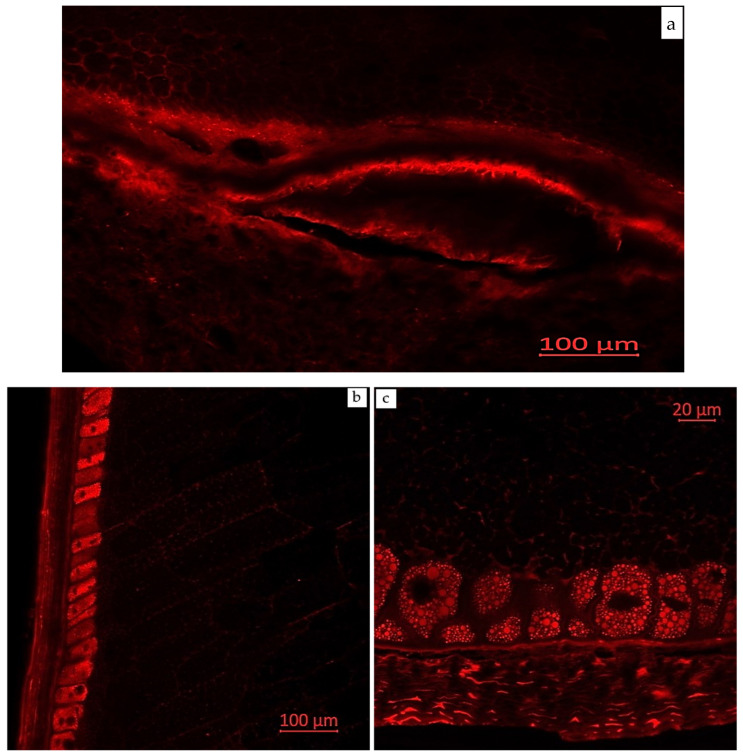
Monospectral images of corn grains (*Zea mays* L., hybrid *Pri-15-7-16*): (**a**) longitudinal section of the seed base (near the attachment point to the cob), 20× magnification; (**b**) longitudinal section, 20× magnification; and (**c**) transverse section of the distal edge of the grain, 63× magnification. Excitation with a blue laser (488 nm) with emission in the range of 620–700 nm (red autofluorescence).

**Figure 6:**
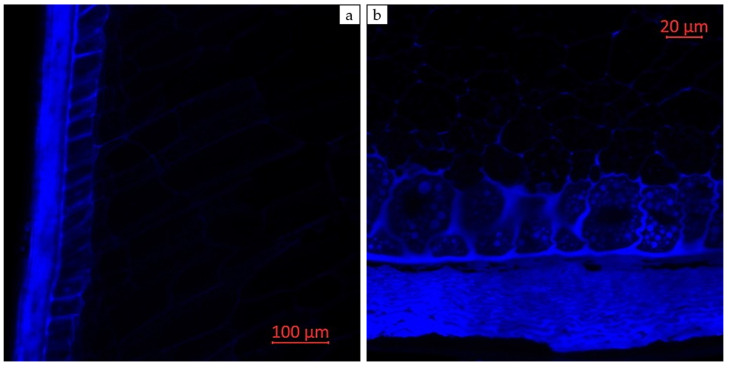
Monospectral images of corn grains (*Zea mays* L., hybrid *Pri-15-7-16*): (**a**) longitudinal section, 20× magnification; and (**b**) transverse section of the distal edge of the grain, 63× magnification. Excitation with a UV (405 nm) with emission in the range of 400–475 nm (blue autofluorescence).

Confocal laser scanning microscopy enables the visualization of various compounds in plant tissues due to their autofluorescent properties. Areas with enhanced autofluorescence indicate regions of high compound concentration. Longitudinal and transverse sections of corn grains (*Zea mays* L., hybrid *Pri-15-7-16*), analyzed using confocal laser scanning microscopy with excitation using a UV laser (405 nm) with emission in the range of 400–475 nm (blue autofluorescence), as well as excitation using a blue laser (488 nm) with emissions in the ranges of 410–545 nm (green autofluorescence), 575–617 nm (yellow autofluorescence), and 620–700 nm (red autofluorescence), are presented in Figure 2, Figure 3, Figure 4, Figure 5 and Figure 6.

## 3. Discussion

A high yield of bioactive compounds from corn grains (*Zea mays* L., hybrid *Pri-15-7-16*) was observed under the following conditions: (1) a pressure of 200 bar and a temperature of 55 °C, yielding 2.2 mg/g with a co-solvent mass fraction of 2%; (2) a pressure of 250 bar and a temperature of 55 °C, yielding 2 mg/g with a co-solvent mass fraction of 2%; and (3) a pressure of 200 bar and a temperature of 50 °C, yielding 2 mg/g with a co-solvent mass fraction of 2%. The supercritical extraction time was 1 h. The optimal extraction conditions were identified as 200 bar and 55 °C, yielding 2.2 mg/g. The data in Table 1 are critical for optimizing the supercritical CO_2_ extraction process for bioactive compound recovery, illustrating how yield varies with pressure and temperature. Higher pressure and temperature parameters may result in lower yields of bioactive compounds during CO_2_ extraction due to several factors, including solvent density, thermal degradation, pressure-dependent solubility, competing temperature effects, extraction time, and compound-specific behavior [31,32,33,34].

The study by Kuś et al. (2018) [27] demonstrated the effectiveness of the central composite rotatable design method for optimizing supercritical CO_2_ extraction conditions from *Populus nigra* L. buds. Pressure and temperature significantly affected extract yield, phenolic compound content, and antioxidant activity. The optimal conditions (30 MPa, 60 °C) maximized the yield and concentration of bioactive flavonoids, making these parameters applicable for obtaining extracts with enhanced levels of target compounds for pharmaceutical use [27]. Another study by Kuś et al. (2018) [25] confirmed the effectiveness of supercritical CO_2_ extraction under optimized conditions (30 MPa, 60 °C) for obtaining bioactive phytochemicals from black poplar (*Populus nigra* L.) buds. The extracts, rich in both volatile and non-volatile bioactive compounds, may provide synergistic effects and enhanced biological activity due to complementary mechanisms of action [25,27].

The study by Villacís-Chiriboga et al. (2021) [34] evaluated the supercritical CO_2_ extraction of bioactive compounds from mango by-products. The research optimized extraction conditions for carotenoids and assessed the co-extraction of phenolics. The optimal conditions were 55 °C, 35 MPa, and 20% ethanol, with β-carotene as the main carotenoid. Phenolic profiles varied between varieties, and the peel contained up to 4.1 times more bioactives than the pulp. Supercritical CO_2_ extraction proved promising for isolating valuable compounds from mango waste, highlighting its potential in sustainable food processing [34].

The study by Molino et al. (2019) [33] investigated the supercritical CO_2_ extraction of β-carotene and fatty acids from *Dunaliella salina* microalgae. The research evaluated the effects of mechanical pre-treatment, biomass loading, pressure, temperature, CO_2_ flow rate, and extraction time. Optimal conditions for β-carotene extraction were 400 bar, 65 °C, and a CO_2_ flow rate of 14.48 g/min, yielding a 25.48% recovery. For fatty acids, the maximum recovery (8.47 mg/g) occurred at 550 bar, 75 °C, and a CO_2_ flow rate of 14.48 g/min. Lower biomass loading (2.45 g) and shorter extraction time (30 min) favored maximum extraction of both compounds [33].

Pimentel-Moral et al. (2019) [32] investigated supercritical CO_2_ extraction of bioactive compounds from *Hibiscus sabdariffa*. Using Response Surface Methodology, they evaluated the effects of temperature, pressure, and co-solvent percentage on extraction efficiency. Optimal conditions for maximum phytochemical content were 50 °C, 250 bar, and 16.7% ethanol. Supercritical CO_2_ is a suitable and selective technique for maximizing the extraction of phytochemical compounds, demonstrating its potential as a green extraction method.

Several studies have investigated supercritical CO_2_ extraction of bioactive compounds from corn grains and by-products, exploring various experimental conditions to optimize extraction yields. Monroy et al. (2016) [38] studied supercritical CO_2_ extraction of phenolic compounds from purple corn ears (*Zea mays* L.), using ethanol and water as co-solvents. They found that pressure, temperature, and co-solvent concentration significantly influenced extraction yields. By evaluating various co-solvent combinations, the study optimized the process, revealing that purple corn ear extract is rich in anthocyanins, phenolic compounds, and flavonoids, exhibiting high antioxidant activity [38].

Marinho et al. (2019) [37] focused on the supercritical CO_2_ extraction of corn germ oil, investigating the effects of temperature (45–85 °C) and pressure (15–25 MPa) on extraction yields. Their results indicated that yields increased with rising pressure at each temperature but decreased with increasing temperature, highlighting the importance of careful parameter control. Additionally, the antioxidant activity of extracts obtained via supercritical CO_2_ extraction was higher than that of extracts obtained through conventional Soxhlet extraction [37].

Overall, studies have shown that supercritical CO_2_ extraction is an effective method for isolating bioactive compounds from various plant materials. This technique offers advantages such as reduced solvent consumption, preservation of sensitive compounds, and the ability to extract both polar and non-polar substances by adjusting parameters such as temperature, pressure, and co-solvent use [26,28,29,30,35,36,39]. However, optimizing the extraction process requires balancing temperature, pressure, and extraction time based on the specific target compounds and plant materials.

In the CO_2_ extracts of corn grains (*Zea mays* L.), 44 compounds were identified—30 from the polyphenol group, including flavones, flavonols, flavan-3-ols, anthocyanidins, and polyphenolic acids, and 14 from other chemical groups, including fatty acids and amino acids. All identified compounds, along with their chemical formulas, molar masses, calculated and observed *m/z* values, and other spectrometric data, are presented in Table A1, Appendix A. The chemical compounds were identified by comparing their retention indices, mass spectra, and mass spectrometry fragmentation patterns with a home-library database created by the Institute of Biotechnology, Bioengineering, and Food Systems at the Advanced Engineering School, Far Eastern Federal University (Russia). This database was built using data from various spectroscopic techniques, including nuclear magnetic resonance, ultraviolet spectroscopy, and mass spectrometry, as well as data from the updated scientific literature.

Fluorescence imaging techniques offer two main advantages over other methods: greater sensitivity and selectivity, owing to the unique properties of autofluorescent molecules that are excited by specific wavelengths and emit light at distinct wavelengths. By utilizing the autofluorescent properties of many plant compounds, fluorescent images can be captured with minimal tissue preparation and, importantly, without the need for labeling [40,41]. The method used in this study is effective for analyzing the distribution of polyphenolic compounds with autofluorescent properties in grains and legume seeds [10,42,43]. This approach allows for the study of plant morphology and the characterization of biologically active phytochemicals using a cost-effective and fast technique.

Figure 2 shows longitudinal and transverse sections of corn grains (*Zea mays* L., hybrid *Pri-15-7-16*) under different fluorescence modes: excitation with a UV laser (405 nm) with emission in the range of 400–475 nm (blue autofluorescence), and excitation with a blue laser (488 nm) with emission in the ranges of 410–545 nm (green autofluorescence) and 575–617 nm (yellow autofluorescence). Image analysis revealed that the pericarp and aleurone layer cell walls are enriched with autofluorescent compounds, consistent with the data from Razgonova et al. (2022) [10].

In plant cell walls, only certain components fluoresce under specific excitation wavelengths. Polysaccharides do not exhibit fluorescence, whereas phenolic compounds, particularly hydroxycinnamic acids and lignin, are the primary natural fluorophores. Hydroxycinnamic acids emit blue fluorescence under UV excitation, while lignin, excited by both UV and visible light, emits blue, green, and red fluorescence. The nature of phenolic compounds, their varying relative proportions, and environmental factors (such as pH and the presence of quenching molecules) lead to different tissue fluorescence responses, which can be interpreted as a tissue’s fluorescent signature [40,44,45,46,47].

Figure 3 presents longitudinal and transverse sections of corn grains (*Zea mays* L., hybrid *Pri-15-7-16*) in a monospectrum, with excitation using a blue laser (488 nm) and emission in the range of 410–545 nm. The green autofluorescence observed in this spectrum may indicate the presence of a significant amount of flavonoid compounds [42,48,49,50], such as flavins and flavonols (myricetin, quercetin, kaempferol, and their derivatives), as confirmed by our mass spectrometric studies. Blue and green autofluorescence were most pronounced in the outer layer of the cotyledons in the seeds of three different soybean species (*G. soja*, *G. gracilis*, and *G. max*). This distribution of phenolic compounds in the outer seed layers may serve a protective function during seed development and against environmental factors [42].

Figure 4 presents longitudinal and transverse sections of corn grains (*Zea mays* L., hybrid *Pri-15-7-16*) in a monospectrum, with excitation using a blue laser (488 nm) and emission in the range of 575–617 nm (yellow autofluorescence). Berger et al. (2021) [40] studied the fluorescence signature of cell walls in maize forage stem sections using multispectral autofluorescence visualization to detect phenolic compounds after UV and visible excitations. UV-induced fluorescence intensity in the rind and nearby parenchyma was associated with the amount of p-coumaric acid, while ferulic acid levels were mainly correlated with the parenchyma near the rind. The study also found that higher lignin content led to increased lignin fluorescence across all tissues, linking yellow and blue fluorescence to lignin and phenolic compounds [40].

Figure 5 shows the longitudinal and transverse sections of corn grains (*Zea mays* L., hybrid *Pri-15-7-16*) in a monospectrum, excitation with a blue laser (488 nm) and emission in the range of 620–700 nm (red autofluorescence). Emission in the red spectrum is mainly due to the presence of various polyphenolic compounds, including anthocyanins and anthocyanidins [51,52,53]. According to mass spectrometric studies, anthocyanidins were identified in the corn grain extracts. The study by Razgonova et al. (2022) [42] examined the spatial distribution of phenolic compounds in the seeds of three different soybean species (*G. soja*, *G. gracilis*, and *G. max*). Red autofluorescence (excitation with a blue laser at 488 nm, emission in the range of 620–700 nm) was strongly correlated with soybean seed color. Black-seeded soybean varieties exhibited the brightest red fluorescence, yellow-seeded varieties showed the weakest, and brown-seeded varieties displayed scattered red fluorescence. As the authors suggested, this fluorescence was linked to anthocyanin content, which was responsible for the black color of the seed coat in legumes [42].

Figure 6 shows the longitudinal and transverse sections of corn grains (*Zea mays* L., hybrid *Pri-15-7-16*) in a monospectrum, with excitation using a UV laser (405 nm) and emission in the range of 400–475 nm (blue autofluorescence). Blue autofluorescence in plants is primarily attributed to the presence of phenolic compounds, particularly lignin and hydroxycinnamic acids [40,41,54,55,56]. The main fluorescent component is ferulic acid, though other hydroxycinnamic acids, such as p-coumaric and caffeic acids, may also contribute to blue fluorescence under UV excitation [55]. Lignin is a well-known source of blue fluorescence in plants [56,57]. However, due to the presence of multiple types of fluorophores within its molecule, lignin exhibits a broad emission range and can be observed under both UV and visible light excitation [40,45,58].

As seen in the monospectral images, the endosperm cell walls exhibit very low blue fluorescence due to their minimal content of fluorescent phenolic compounds, which is consistent with previous studies [10,59]. In addition, the pericarp of *Zea mays* grains has been reported to contain a total phenolic content 30–34 times higher than that of the endosperm [60]. According to Razgonova et al. (2022) [10], who studied the distribution of polyphenolic compounds in *Zea mays* L. var. *Pioneer* grains using laser microscopy, the aleurone layer of the grain was enriched with polyphenolic substances emitting blue autofluorescence. However, as previously reported, since the aleurone layer does not contain lignin [57], the authors concluded that the observed blue fluorescence may be due to the presence of significant amounts of hydroxycinnamic acids, such as ferulic and coumaric acids [61,62].

Fluorescence microscopy data revealed the most probable localizations of polyphenolic compounds. However, it is important to note that this method has certain limitations: while effective for determining the spatial arrangement of chemical substance groups, it does not allow for the identification of individual compounds. These methodological limitations should be considered when interpreting the results.

For a more accurate interpretation of autofluorescence results in plant cell walls, further research on the quantitative analysis of bioactive compounds would be beneficial. Additionally, other methods, such as Wiesner’s or Maule’s stains or other selective dyes for lignin, could be applied. It may also be advantageous to consider additional microspectroscopic techniques, such as Raman or infrared imaging, to further localize phenolic compounds alongside cell wall polysaccharides [40].

## 4. Materials and Methods

The object of this study was corn grains (*Zea mays* L., hybrid *Pri-15-7-16*) obtained from the collection of the Federal Scientific Center of Agricultural Biotechnology of the Far East named after A.K. Chaika, Ussuriysk, Russia. These grains were grown and harvested during August–September 2022 in fields located near Ussuriysk, 120 km from Vladivostok, Russia, on the Ussuri-Khankai plain. This region is characterized by a warm and humid climate with harsh winters. The hydrothermal coefficient varies from 1.6 to 2.0 (excessively humid). The hybrid corn (*Zea mays* L.) was assigned its name by the Federal Scientific Center of Agricultural Biotechnology of the Far East named after A.K. Chaika according to their classification. These varieties are included in the State Register of Breeding Achievements of the Russian Federation and are approved for use. The corn grains were pre-ground in a universal mill to a particle size of 2–3 mm.

### 4.1. Fractional Maceration

To obtain highly concentrated extracts, the fractional maceration technique was applied. The total amount of extractant (methyl alcohol of reagent grade ≥ 99.5%) was divided into three parts and successively infused with portions of the plant material: first, with the first portion; then, with the second; and finally, with the third. The infusion time for each part was seven days at room temperature.

### 4.2. Supercritical CO_2_ Extraction

Supercritical CO_2_ extraction was performed using the SFC-500 supercritical fluid chromatography system (Thar Instruments, Inc., Pittsburgh, PA, USA). The system included a supercritical extraction compressor for compressing CO_2_ to the required pressure, a CO_2_ flow meter (Siemens, München, Germany) to measure the CO_2_ input, a co-solvent pump for supplying the co-solvent, and a 1 L extraction vessel. The extraction vessel was heated with a hot jacket controlled by a thermostat (±1 °C) while pressure was regulated by a dosing valve. Ground plant matrices (100 g) were loaded into the extractor with a CO_2_ flow rate of 250 g/min. Supercritical extracts were obtained under various CO_2_ pressures (50–400 bar) and temperatures (31–70 °C), using minimal amounts of ethanol as a co-solvent. Extracts were collected in a separator connected to the dosing valve and maintained in a circulation bath at 0 °C. The extraction time began after achieving working pressure and equilibrium flow with CO_2_ pressure and temperature optimized to maximize product yield. This supercritical CO_2_ extraction method was tested on various plant matrices.

### 4.3. Liquid Chromatography

High-performance liquid chromatography (HPLC) was performed using a Shimadzu LC-20 Prominence HPLC system (Shimadzu, Kyoto, Japan) equipped with a UV sensor and a C18 silica reverse-phase column (4.6 × 150 mm, particle size: 2.7 µm) for the separation of multi-component mixtures. The gradient elution program with two mobile phases (A: deionized water; B: acetonitrile with 0.1% *v*/*v* formic acid) was as follows: 0–2 min, 0% B; 2–50 min, 0–100% B; and control washing 50–60 min, 100% B. The entire HPLC analysis was performed using a UV–vis detector SPD-20A (Shimadzu, Kyoto, Japan) at wavelengths of 230 and 330 nm. The column temperature was maintained at 50 °C, with a total flow rate of 0.25 mL min^−1^. The injection volume was 10 µL. Additionally, liquid chromatograph was combined with a mass spectrometric ion trap AmaZon SL (Bruker Daltoniks, Bremen, Germany) for compound identification.

### 4.4. Mass Spectrometry

Mass spectrometry (MS) analysis was performed using an ion trap AmaZon SL (Bruker Daltoniks, Bremen, Germany) equipped with an electrospray ionization (ESI) source operating in both negative and positive ion modes. The optimized parameters were as follows: ionization source temperature: 70 °C, gas flow: 4 L/min, nebulizer gas (atomizer) pressure: 7.3 psi, capillary voltage: 4500 V, endplate bend voltage: 1500 V, fragmentor voltage: 280 V, and collision energy: 60 eV. The ion trap was used within the scanning range of *m*/*z* 100–1700 for MS and MS/MS. The acquisition rate was one spectrum per second for MS and two spectra per second for MS/MS. A four-stage ion separation mode (MS/MS mode) was implemented. Data collection was controlled by Hystar Data Analysis 4.1 software (Bruker Daltoniks, Bremen, Germany) and synchronized with the Shimadzu LC-20 Prominence HPLC system. All experiments were repeated three times.

The chemical compounds were identified by comparing their retention index, mass spectra, and mass spectrometry fragmentation patterns with data from a home-library database created by the Institute of Biotechnology, Bioengineering, and Food Systems at the Advanced Engineering School, Far Eastern Federal University (Russia), as well as other databases (MS2T, MassBank, HMDB).

### 4.5. Confocal Laser Scanning Microscopy

Confocal laser scanning microscopy was performed according to the method described by Razgonova et al. (2022) [10]. Before microscopic examination, longitudinal and transverse sections of dry, untreated corn grains were prepared using an MS-2 sliding microtome (Tochmedpribor, Harkiv, Ukraine). The obtained slices of corn grains were placed on a microscope slide with immersion oil to reduce light refraction caused by air gaps. The autofluorescence parameters of the corn grain slices were determined using an inverted confocal laser scanning microscope (LSM 800, Carl Zeiss Microscopy GmbH, Jena, Germany). The autofluorescence spectrum was selected using the λ-scanning mode of the confocal microscope, which allows for determining the emission maximum in a specific sample and obtaining spectra. A λ-experiment was conducted with excitation using lasers of 405 and 488 nm in succession, recording the emission in the range from 400 to 700 nm with a step of 5 nm. The following fluorescence maxima were identified: excitation with a UV laser (405 nm, solid-state, diode, 5 mW) with emission in the range of 400–475 nm (blue); excitation with a blue laser (488 nm, solid-state, diode, 10 mW) with emissions in the ranges of 410–545 nm (green), 575–617 nm (yellow), and 620–700 nm (red). Images were obtained using Plan-Apochromat 20×/0.8 M27 and Plan-Apochromat 63×/1.40 Oil DIC M27 objectives. ZEN 2.1 software (Carl Zeiss Microscopy GmbH, Germany) was used for image acquisition.

## 5. Conclusions

The research demonstrated the effectiveness of supercritical CO_2_ extraction in isolating bioactive compounds from corn grains (*Zea mays* L., hybrid *Pri-15-7-16*). The optimal extraction conditions were determined to be a pressure of 200 bar and a temperature of 55 °C, yielding 2.20 mg/g of bioactive compounds. These results highlight the potential of supercritical CO_2_ extraction as an efficient method for obtaining valuable bioactive compounds. However, optimizing the extraction process requires balancing temperature, pressure, and extraction time based on the specific target compounds and plant materials.

Metabolomic analysis identified a diverse array of 44 compounds, including 30 polyphenols from various subgroups—flavones, flavan-3-ols, flavonols, polyphenolic acids, and anthocyanidins—as well as compounds from other chemical groups, such as amino acids and fatty acids. This diversity underscores the potential of corn grains as a rich source of bioactive compounds.

Additionally, the distribution of bioactive compounds in corn grain tissues was visualized using confocal laser scanning microscopy, providing insights into their localization. Image analysis showed that the pericarp and aleurone layer cell walls were rich in autofluorescent compounds, while the endosperm cell walls exhibited low autofluorescence. The ability of phenolic compounds to emit autofluorescence enables their detection and analysis in plant cell walls without additional labeling. Understanding the distribution of polyphenolic compounds in plant tissues will aid in developing methods for their direct extraction and further applications in the food, feed, pharmaceutical, and cosmetic industries.

## Figures and Tables

**Table 1:** Yield of bioactive compounds (mg/g) from corn grains (*Zea mays* L., hybrid *Pri-15-7-16*) under various pressure and temperature conditions during supercritical CO_2_ extraction.

Temperature, °C	50 bar	100 bar	150 bar	200 bar	250 bar
31	0.30	0.40	0.50	0.56	1.00
40	0.40	0.40	0.70	1.50	1.30
45	0.50	0.50	0.90	1.60	1.50
50	0.65	0.65	1.00	2.00	1.80
55	0.70	1.20	1.50	2.20	2.00
60	1.00	1.00	1.40	1.80	1.50

## Data Availability

The data presented in the current study are available in the article.

## Appendix A

**Table A1:** Bioactive compounds identified in supercritical CO_2_ extracts of corn grains (*Zea mays* L.) by tandem mass spectrometry (MS/MS) in positive and negative ionization modes.

	Classes of Compounds	Identified Chemical Compounds	Chemical Formula	Molar Mass	Ion Adduct [M−H]^−^	Ion Adduct [M+H]^+^	First-Order Fragmentation MS/MS	Second-Order Fragmentation MS/MS	Third-Order Fragmentation MS/MS	Sources
**Polyphenolic Compounds**										
1	Polyphenolic acid	Caffeic acid derivative	C_16_H_18_O_9_Na	377.2985	377		341; 215	179; 113		[63]
2	Polyphenolic acid	Caffeoylmalic acid	C_13_H_12_O_8_	296.2296	295		277; 171	233; 113		[64,65]
3	Polyphenolic acid	3,4-Diacetoxybenzoic acid	C_10_H_11_O_6_	238.1935	237		119			[64,66]
4	Polyphenolic acid	Hydroxy methoxy dimethylbenzoic acid	C_10_H_12_O_4_	196.1999		197	177; 153	125		[67]
5	Polyphenolic acid	Hydroxyferulic acid	C_10_H_10_O_5_	210.1834		211	193; 125			[68]
6	Polyphenolic acid	Caffeic acid [(2E)-3-(3,4-Dihydroxyphenyl) acrylic acid]	C_9_H_8_O_4_	180.1574		181	135	119		[69,70,71]
7	Stilbene	Resveratrol [trans-Resveratrol; 3,4’,5-Trihydroxystilbene; Stilbentriol]	C_14_H_12_O_3_	228.2433		229	209	163	146	[67,72]
8	Flavan-3-ol	Epiafzelechin [(epi)Afzelechin]	C_15_H_14_O_5_	274.2687		275	245; 176	175		[67,73,74]
9	Flavonol	Kaempferol [3,5,7-Trihydroxy-2-(4-hydro- xyphenyl)-4H-chromen-4-one]	C_15_H_10_O_6_	286.2363	285		185; 117; 257	117		[64,68,75,76,77]
10	Flavan-3-ol	Catechin [D-Catechol]	C_15_H_14_O_6_	290.2681		291	261; 189	173; 242	191; 143	[67,72,78,79,80]
11	Flavan-3-ol	(epi)catechin	C_15_H_14_O_6_	290.2681		291	261; 173	243; 173		[67,72,80]
12	Flavonol	Quercetin	C_15_H_10_O_7_	302.2357		303	275; 245; 203; 175	175		[63,64,75,78]
13	Flavan-3-ol	Gallocatechin [+ (-) Gallocatechin]	C_15_H_14_O_7_	306.2675		307	277; 207	207; 159		[67,78,79]
14	Flavonol	Myricetin	C_15_H_10_O_8_	318.2351		319	291; 219; 174	259; 191	243; 161	[71,72,77,81,82]
15	Flavone	Cirsiliol	C_17_H_14_O_7_	330.2889	329		229; 171; 293	211; 155	183	[83]
16	Flavone	5,7-Dimetoxy-3,3’,4’-trihydroxyflavone	C_17_H_14_O_7_	330.2889		331	315; 270	313	285; 257	[84]
17	Flavone	Luteolin 7,3’-disulphate	C_15_H_10_O_12_S_2_	446.3627		447	287	152		[85]
18	Flavone	Apigenin 7-sulfate	C_15_H_10_O_8_S	350.3001		351	337; 308	308; 291		[67,86]
19	Lignan	Matairesinol [(-)-Matairesinol; Artigenin Congener]	C_20_H_22_O_6_	358.3851		359	324; 289; 127	144	127	[87]
20	Flavan-3-ol derivative	Epiafzelechin 3-*O*-gallate	C_22_H_18_O_9_	426.3729		427	301; 171; 382	171		[80]
21	Flavone	Apigenin-*C*-hexoside	C_21_H_20_O_10_	432.3775		433	418; 314; 265; 219; 155	257; 169		[88]
22	Anthocyanidin	Pelargonidin-3-*O*-glucoside (callistephin)	C_21_H_21_O_10_	433.3854		433	271; 185	253; 121	235	[89,90]
23	Anthocyanidin	Cyanidin-3-*O*-glucoside [Cyanidin 3-*O*-beta-D-Glucoside; Kuromarin]	C_21_H_21_O_11_+	449.3848	447		285	199		[90,91,92]
24	Flavone	Luteolin-7-*O*-beta-glucuronide	C_21_H_18_O_12_	462.3604		463	447; 395; 359; 285; 199; 149	287; 199		[93,94]
25	Flavonol	Kaempferol-3-*O*-glucuronide	C_21_H_18_O_12_	462.3604		463	287; 198	269; 198		[65,67,75]
26	Anthocyanidin	Delphinidin malonyl hexoside	C_24_H_23_O_15_	551.4304		551	465; 287; 185	287; 115		[67]
27	Flavone	Chrysoeriol *C*-hexoside-*C*-pentoside	C_27_H_30_O_15_	594.5181		595	578; 536; 509; 425	294		[66]
28	Flavonol	Quercetin 3,4’-di-*O*-beta-glucopyranoside [Quercetin diglucoside]	C_27_H_30_O_17_	626.5169		627	465	447; 405; 303		[64,77,92]
29	Flavone	Tricin trimethyl ether 7-*O*-hexosyl-hexoside	C_30_H_36_O_17_	668.5966		669	345; 387; 283			[95]
30	Flavan-3-ol	(Epi)fisetinidol-(epi)catechin-A-(epi)fisetinidol	C_45_H_36_O_16_	832.7577	831		721; 693; 609; 575; 537; 506			[96]
**Compounds of Other Chemical Groups**										
31	Amino acid	L-Lysine	C_6_H_14_N_2_O_2_	146.1876		147	119			[76]
32	Amino acid	L-threanine	C_7_H_14_N_2_O_3_	174.1977		175	159			[80]
33	Amino acid	L-Tryptophan [Tryptophan; (S)-Tryptophan]	C_11_H_12_N_2_O_2_	204.2252		205	161; 159	143		[80,97]
34	Omega-5 fatty acid	Myristoleic acid [Cis-9-Tetradecanoic acid]	C_14_H_26_O_2_	226.3550		227	209; 168	127		[67]
35	Medium-chain fatty acid	Hydroxy dodecanoic acid	C_12_H_22_O_5_	246.3001		247	238	203	174	[67]
36	Omega-3 fatty acid	Stearidonic acid [6,9,12,15-Octadecatetraenoic acid; Moroctic acid]	C_18_H_28_O_2_	276.4137		277	259; 177	177		[67,75,98]
37	Omega-3 fatty acid	Linolenic acid (Alpha-Linolenic acid; Linolenate)	C_18_H_30_O_2_	278.4296		279	243; 173	173	131	[98,99]
38	Diterpenoid	Isocryptotanshinone II	C_19_H_20_O_3_	296.3603		297	279; 197	173		[98]
39	Alpha-omega Dicarboxylic acid	Octadecanedioic acid [1,16-Hexadecanedicarboxylic acid]	C_18_H_34_O_4_	314.4602	313		295; 183	293; 179	275; 177	[67]
40	Unsaturated fatty acid	Oxo-eicosatetraenoic acid	C_20_H_30_O_3_	318.4504		319	301	186		[67]
41	Oxylipin	13-Trihydroxy-Octadecenoic acid [THODE]	C_18_H_34_O_5_	330.4596	329		171; 211; 293	153		[100,101]
42	Oxylipin	9,12,13-Trihydroxy-*trans*-10-octadecenoic acid	C_18_H_34_O_5_	330.4596	329		171; 229	127		[64]
43	Carotenoid	(all-E)-lutein 3’-*O*-myristate	C_40_H_54_O	550.8562		551	533; 505; 469;429; 373; 345	453; 410		[102]
44	Unsaturated fatty acid	Eicosatetraenedioic acid	C_20_H_30_O_4_	334.4498		335	321; 124	291		[67]
